# Typhoid Fever in Chile

**DOI:** 10.4269/ajtmh.18-0677

**Published:** 2018-12

**Authors:** Felipe Cabello

**Affiliations:** Department of Microbiology and Immunology; New York Medical College; Valhall, NY; E-mail: felipe_cabello@nymc.edu

To the Editor:

A recent article in this Journal from Marco et al., “Typhoid Fever in Chile 1969–2012: Analysis of an Epidemic and Its Control” has reanalyzed a major typhoid fever epidemic and its control in Chile in the latter part of the 20^th^ century.^1^ Such reexaminations are relevant because typhoid fever continues to be an important health problem in most of the developing world, with an annual mortality of roughly 200,000, and because effective treatment is imperiled by the emergence of extensively drug-resistant (XDR) *Salmonella enterica* serovar Typhi.

Marco et al.^1^ conclude that the Chilean epidemic was caused by contaminated vegetable crops rather than by contaminated municipal drinking water. The reasoning used by Marco et al. to discard the conclusions of our previously published analysis of the Chilean urban typhoid epidemic is weak.^2^

We and others have identified clear failures in water sanitation documented in the scientific and lay press and in technical reports at the time of this epidemic.^2,3^ These failures included lack of chlorination, faulty filtration, and suspension of drinking water services for nonpayment of fees at the plant providing drinking water to most of the population of Santiago.^2,3^

*Salmonella* Typhi was never isolated from drinking water, as mentioned by Marco et al., but they neglect to mention that it was also never isolated from vegetables. For that matter, vegetables were never found to be a risk factor for the disease.^2,4,5^ Furthermore, and contrary to the statements of Marco et al.,^1^ our work^6^ and that of Thong et al.^7^ support both the hypothesis of vegetable vehicles for *S*. Typhi transmission and the hypothesis that the epidemic was the result of suboptimally prepared drinking water because water used for drinking and irrigation can be contaminated by carriers and typhoid fever patients as well as by raw sewage containing the pathogen.^6,7^ In both cases, the same strains will be present in patients and in the environment, a conclusion stated by us^6^ and by Thong et al.^7^

In addition, the very low frequency of typhoid fever among infants and its benign clinical presentation in this population^1,8^ also appear to undermine the authors’ rationale that deficient drinking water would have a severe impact on infant mortality. In summary, the three rationales put forward by the authors fail to undermine our published data-based hypothesis on the role of defective drinking water sanitation in the typhoid fever epidemic in Chile.

In industrialized and developing countries,^9–11^ urban epidemics of typhoid, with sudden beginnings and thousands of cases affecting all ages, have been the result of shortcomings in water sanitation, including defective chlorination and filtration, anastomosis between drinking water and sewer lines, and suspension of service, all these factors occasionally linked to excessive rainfall and floods.^1,9–11^ The putative role of contaminated vegetables as a cause of this epidemic is further weakened by the failure by the authors to present any evidence for large increases in vegetable irrigation with sewage potentially contaminated with *S*. Typhi and with surges in vegetable consumption by the populace of Santiago that could explain the abrupt peaks of the disease in 1977 and 1982. The abrupt fluctuations in morbidity in this epidemic curve are similar to those found in the description of typhoid epidemics generated by defects in water sanitation in many localities in the past and present.^9−11^ It may well be that many factors, including vegetable contamination with *S*. Typhi, were responsible for endemic typhoid in Chile preceding this epidemic, but the evidence at hand strongly indicates that a novel and decisive trigger was introduced in 1977, and that this trigger was defective water sanitation.^1,2^ Correction of the shortcomings in water purification subsequent to their identification, subsidence of the economic crisis, and some of the additional factors mentioned by Marco et al. may have been responsible for abatement of this epidemic and endemic typhoid in Chile.^2,10,12^

Typhoid fever epidemics, particularly those produced by XDR *S*. Typhi, appear to be increasing worldwide. Situations like the Chilean epidemic, which lasted 10 years, with approximately 70,000 cases and more than 500 deaths, need to be prevented at all costs. Careful analyses of the causes of epidemics are essential, and when available, need to be taken into account by subsequent studies.
